# Association of Genetic Profile with Muscle Mass Gain and Muscle Injury Prevention in Professional Football Players after Creatine Supplementation

**DOI:** 10.3390/nu16152511

**Published:** 2024-08-01

**Authors:** David Varillas-Delgado

**Affiliations:** 1 Exercise and Sport Science, Faculty of Health Sciences, Universidad Francisco de Vitoria, 28223 Pozuelo, Spain; david.varillas@ufv.es; 2 SPORTNOMICS S.L., 28922 Madrid, Spain

**Keywords:** creatine supplementation, football, genes, single-nucleotide polymorphism, muscle mass, muscle performance, injuries

## Abstract

Background: In recent years, the study of creatine supplementation in professional athletes has been of great interest. However, the genetics involved in response to supplementation is unknown. The aim of this study was to analyse, for the first time, the relationship between muscle performance-related genes and the risk of an increased body mass index (BMI) and muscle mass and a decrease in fat mass in professional football players after creatine supplementation. Methods: For this longitudinal study, one hundred and sixty-one men’s professional football players were recruited. The polymorphisms *ACE* I/D, *ACTN3* c.1729C>T, *AMPD1* c.34C>T, *CKM* c.*800A>G, and *MLCK* (c.49C>T and c.37885C>A) were genotyped using Single-Nucleotide Primer Extension (SNPE). To assess the combined impact of these six polymorphisms, a total genotype score (TGS) was calculated. The creatine supplementation protocol consisted of 20 g/day of creatine monohydrate for 5 days (loading dose) and 3–5 g/day for 7 weeks (maintenance dose). Anthropometric characteristics (body mass index (BMI), fat, and muscle mass) were recorded before and after the creatine supplementation protocol. Characteristics of non-contact muscle injuries during the 2022/2023 season were classified according to a consensus statement for injury recording. The results showed that the allelic frequencies of *ACE* and *AMPD1* differed between responders and non-responders in muscle mass increase (all *p* < 0.05). Players with a TGS exceeding 54.16 a.u. had an odds ratio (OR) of 2.985 (95%CI: 1.560–5.711; *p* = 0.001) for muscle mass increase. By contrast, those with a TGS below 54.16 a.u. had an OR of 9.385 (95%CI: 4.535–19.425; *p* < 0.001) for suffering non-contact muscle injuries during the season. Conclusions: The increase in BMI and muscle mass in response to creatine supplementation in professional football players was influenced by a TGS derived from the combination of favourable genotypes linked to muscle performance. The CC genotype and C allele of *AMPD1* were particularly associated with a higher likelihood of muscle mass increase under creatine supplementation in this group of professional football players.

## 1. Introduction

Creatine, also known as methyl guanidine-acetic acid (C_4_H_9_N_3_O_2_), is a natural compound produced endogenously in the body and obtained through diet, especially from red meat (pork, venison, and elk) [1]. It is estimated that the daily requirement of creatine for a 70 kg male is 2 g/day and that up to half may be obtained from a typical omnivorous diet, the remaining amount being produced by the body [2,3]. Creatine is one of the most studied and scientifically supported supplements in the field of sports performance and muscle mass gain inducing an increase in the body mass index (BMI) [4,5,6]. When supplemented with exogenous creatine, intramuscular and brain stores of creatine and its phosphorylated form—phosphocreatine (PCr)—increased [7,8]. PCr is used to regenerate adenosine triphosphate (ATP), which is the main source of energy for explosive activities [6] and plays a crucial role in energy production during high-intensity, short-duration exercise, such as weightlifting. Also, it has been suggested that creatine supplementation reduces muscle damage following intense exercise and improves subsequent recovery, as well as attenuating oxidative stress and the inflammatory response [9,10]. Moreover, several studies suggest that the increase in muscle PCr accelerates calcium homeostasis, due to the phosphorylation of adenosine diphosphate (ADP) to ATP, reducing cytosolic levels of this mineral and increasing them in the sarcoplasmic reticulum, which leads to a reduction in muscle damage [9,11,12].

Previous research has established that creatine supplementation increases muscle mass, although this seems to be greater when combined with strength exercise [13,14,15]. Several processes have been proposed to explain the mechanism by which creatine supplementation influences lean mass. (i) Creatine increases cellular hydration status (exhibiting osmotic properties), which may serve as an anabolic stimulus (mediated by specific protein kinases and signalling pathways) for protein synthesis. Creatine increases cellular hydration, enhancing cell volume and acting as an anabolic stimulus. It activates protein kinases and pathways like mTOR, crucial for protein synthesis and muscle growth [13]. (ii) Creatine is able to modulate the insulin-like growth factor and myogenic transcription factors, which stimulate satellite cell activation, differentiation, and proliferation, which may ultimately increase the ability to synthesise muscle proteins. Creatine modulates the insulin-like growth factor (IGF-1) and myogenic transcription factors, stimulating satellite cell activation, differentiation, and proliferation, thereby promoting muscle protein synthesis and repair [16], and (iii) creatine appears to decrease markers of muscle protein catabolism in the body, as well as inflammation and oxidative stress, leading to greater muscle mass accumulation over time [17].

To date, the efficacy of ergogenic supplementation on safety and sports performance has not been fully demonstrated [2,18]. However, many studies advocate the use of supplements such as caffeine, β-alanine, and creatine, which can have a positive influence on performance, especially in football [19,20]. A high endurance capacity is a prerequisite for optimal performance in matches, especially if extra time is played [19]. Another effect of creatine supplementation is the reduction in muscle damage after exercise (24 to 96 h after exercise). A useful biomarker in these cases is creatine kinase (CK), a marker of muscle damage [21].

It is crucial to note that the efficacy of creatine supplementation could vary between individuals [22]. Some athletes may experience a more pronounced response in BMI and muscle mass gain with the same protocol, with male athletes being better responders than female athletes [13].

Several genetic variants have been associated in recent years with muscle performance, and their relationship to sports performance and the risk of injuries in professional football shows that muscles with higher performance, as defined by genetic profile, are associated with a lower risk of injuries [23,24]. The angiotensin-converting enzyme (*ACE*) I/D (rs4646994) polymorphism has been extensively studied in sports performance and has been shown to be associated with hypertension, with the D allele showing a favourable association with muscle performance [25]. The alpha actinin-3 (*ACTN3*) c.1729C>T (rs1815739) polymorphism is related to muscle fibre composition, with the C allele being associated with power and sprint performance [26] and fast-twitch muscle fibres modulating skeletal muscle response to exercise in athletes [27]. The adenosine monophosphate deaminase isoform 1 (*AMPD1*) c.34C>T (rs17602729) polymorphism is related to muscle metabolism, which is key to athletic performance, showing the T allele as a predictor of cramps, early fatigue, and muscle injury [23,28,29,30]. Muscle-specific creatine kinase (*CKM*) c.*800A>G (rs8111989) and creatine kinase light-chain [MLCK; c.49C>T (rs2700352) and c.37885C>A (rs2849757)] polymorphisms are related to creatine kinase activity, sports performance, muscle damage, and the risk of injuries in professional football players [31,32,33].

While creatine is a well-researched supplement with proven benefits for muscle mass and performance, personalised recommendations remain challenging due to individual differences. More targeted research is needed to establish specific guidelines that consider factors such as gender, age, activity level, and endogenous synthesis capabilities [17,34,35]. Indeed, despite extensive research on genetic markers related to sports performance and muscle metabolism, the association of these genetic markers with the response to creatine supplementation in professional football players has not yet been studied.

Therefore, the aim of this study was to analyse, for the first time, the relationship between muscle performance-related genes, and the probability of increase in BMI and muscle mass and decrease in fat mass in professional football players after creatine supplementation, as well as demonstrate the feasibility of a total genotype score that correlates significantly with muscle injuries during the season. It is hypothesised that genetic variants related to muscle performance could influence the response to creatine supplementation in professional football players, which in turn could reduce the incidence of muscle injuries throughout the season.

## 2. Materials and Methods

This study examined the same cohort of athletes presented in Maestro et al. [23] from the same football clubs. However, this study employed a novel set of analyses to address a new set of hypotheses not previously reported, with injury data recorded during the 2022/2023 season. For the purposes of this study, ‘muscle performance’ is viewed in relation to the rate of injury in accordance with past work of this author [23] and not in the sense of the standard functional assessment. Thus, in this sense, a higher muscle performance is related to a lower rate of injury and vice versa.

### 2.1. Participants

One hundred and sixty-one men’s professional football players from LaLiga Santander and LaLiga Smartbank in Spain were recruited in this longitudinal study during the 2022/2023 season. The baseline characteristics of the professional football players are presented in Table 1.

The inclusion criteria for participants were as follows: (i) professional football players contracted with the first team during the 2022/2023 season, (ii) professional football players following the same creatine supplementation protocol, and (iii) professional football players of Spanish Caucasian descent for at least three generations to avoid ethnicity biases [23,36]. The exclusion criteria were (i) incapacitating injuries preventing football training or matches in the six months prior to the study, (ii) professional football players using other supplements (e.g., iron, glutamine, beta-alanine) during the season, (iii) players who forgot to take their creatine supplements during the protocol period, and (iv) professional women’s football players.

All participants provided written informed consent to partake in this study. The study protocol received approval from the research ethics committee of Francisco de Vitoria University (UFV 32/2020). Participant confidentiality was maintained in accordance with the Declaration of Helsinki 1964 (latest update, 2013).

### 2.2. DNA Sample Collection and Genotyping

Samples were collected during the 2022/2023 season following creatine supplementation. Buccal smears were obtained using SARSTED swabs and stored at 4 °C until genotyping.

DNA extraction was performed at the VIVOLabs laboratory (Madrid, Spain) using the automated QIACube system (QIAGEN, Venlo, The Netherlands), yielding a DNA concentration of 25–40 ng/mL. The DNA was kept in a 100 μL solution at −20 °C until genotyping.

The polymorphisms *ACE* I/D (rs4646994), *ACTN3* c.1729C>T (rs1815739), *AMPD1* c.34C>T (rs17602729), *CKM* c.*800A>G (rs8111989), *MLCK* c.49C>T (rs2700352), and c.37885C>A (rs28497577) were genotyped using single-nucleotide primer extension (SNPE) with the SNaPshot Multiplex Kit (Thermo Fisher Scientific, Waltham, MA, USA). The results of the reactions were analysed using capillary electrophoresis on an ABI3500 unit (Applied Biosystems, Foster City, CA, USA), using GeneMapper 5.0 software (Applied Biosystems, CA, USA) for bioinformatic analysis. Based on previous studies focused on football players, these polymorphisms were selected for their associations with power and muscle strength [37,38], human physical performance [39,40,41,42,43], and susceptibility to muscle damage and injuries [23,31,33].

The genomic location of each polymorphism is presented in Table 2.

### 2.3. Creatine Supplementation Protocol

This research involved monitoring all players adhering to a creatine supplementation protocol, which was initiated at the beginning of the season in July/August 2022, as reported by the medical services of the participating clubs throughout the 2022/2023 season. To be included in the analysis, the protocol of creatine supplementation had to be the same in all players under medical supervision to avoid side effects, and players who did not follow the recommendations or forgot to take creatine on any day of the protocol were excluded from the investigation. For this reason, 14 professional football players were excluded from this study, 9 for side effects (mild stomach discomfort, headache, dehydration, irritability, and muscle cramps) and 5 for forgetting to take creatine during several days of the protocol. The medical staff of each football club administered the protocol, which consisted of a loading dose of around 20 g/day during 5 days followed by a maintenance dose of 3–5 g/day during 7 weeks (equivalent to approximately 0.3 g/kg/day and 0.03 g/kg/day, respectively0), which was previously used for sports performance and football training [22,46]. The supplementation consisted of Creatine Monohydrate (C_4_H_9_N_3_O_2_*H_2_O) (Myprotein, Manchester, UK) in a mixture of the previously shown creatine dose in a shaker with 500 mL of water.

### 2.4. Injury Data Collection

Muscle injury data were collected from the conclusion of the creatine supplementation protocol in October 2022 until the end of the season in May/June 2023. These data included all injuries reported by the medical services of the clubs. For an injury to be included in the analysis, it had to result from football activities during training or competition and involve muscle tears in a lower limb. Injuries caused by collisions with another player or an object were excluded, as these are unlikely to be influenced by the player’s genotype. Each club’s doctor recorded the data on a paper with the player injury questionnaire, which was completed whenever a player required medical attention and then sent to the head of medical services. Injury data collection followed the international consensus statement on procedures for epidemiological studies of football injuries, as recommended by FIFA and the Union of European Football Associations [47,48,49].

### 2.5. Anthropometric Data Collection

Anthropometric data for all professional football players were evaluated by the medical staff at both the start of this study and the conclusion of creatine supplementation, following the guidelines provided by the International Society for Advanced Kinanthropometry (ISAK) [50]. Height (cm) was measured with a SECA 220^®^ rod (Hamburg, Germany), and body mass (kg) was recorded using an Inbody 770^®^ device (Cerritos, CA, USA). Body fat mass was calculated using the equations by Carter, Faulkner, Yuhasz, and Withers, while body muscle mass was estimated using the Lee 2000 equation [51].

Data were collected under basal conditions after a 10–12 h overnight fast, and euhydration status was assessed by measuring urine-specific gravity with a refractometer (MASTER-S28M, Atago Company, Tokyo, Japan).

### 2.6. Polygenic Potential for Muscle Performance

The combined effect of the six polymorphisms was assessed using a total genotype score (TGS), based on the method outlined by Williams and Folland [52]. A genotype score (GS) of 2 was given to the ‘optimal’ genotype for muscle performance, a GS of 1 was assigned to the heterozygous genotype, and a GS of 0 was allocated to the ‘worst’ genotype for muscle performance in football players, as demonstrated in previous studies [23,24]. The GS scores for the six polymorphisms within the cohort of professional football players are detailed in Table 3.

The GS for all genotypes were converted to a scale of 0–100 arbitrary units (a.u) to simplify interpretation. This converted score, known as the TGS, was as follows:

TGS = (GS_AMPD1_ + GS_ACE_ + GS_ACTN3_ + GS_CKM_ + GS_MLCK49_ + GS_MLCK37885_) × 100/12

### 2.7. Statistical Analysis

Statistical analysis was conducted using the Statistical Package for the Social Sciences (SPSS), version 21.0 for Windows (IBM Corp. Released 2012. IBM SPSS Statistics for Windows, Version 21.0. IBM Corp. Armonk, NY, USA).

SNPs disequilibria were assessed using the Hardy–Weinberg Equilibrium (HWE) and the method proposed by Weir and Cockerham [53]. The χ^2^ test was employed to evaluate the probability of having an optimal genotype for these polymorphisms with respect to changes in BMI, fat, and muscle mass, with a fixed α error of 0.05. The genotypic frequencies of the polymorphisms were compared across different anthropometric characteristics using the χ^2^ test with a fixed α of 0.05. Professional football players who showed positive, negative, or no adaptations to creatine supplementation were identified based on the absolute change relative to the smallest worthwhile change (SWC = standard error of the estimate (SEE) × √2 × 1.962) [54]. The effectiveness of the TGS in distinguishing responses in BMI (0 = no, 1 = yes), fat mass (0 = yes, 1 = no), and muscle mass (0 = no, 1 = yes) was evaluated using a receiver operating characteristic (ROC) curve [55]. The area under the ROC curve (AUC) was calculated with 95% confidence intervals (95%CI). Lastly, a binary logistic regression model was used to examine the relationship between TGS and changes in anthropometric characteristics and muscle injuries, with odds ratios (ORs) being computed.

## 3. Results

The polymorphisms analysed met the HWE (all *p* > 0.05; Table 2).

Of the players included, 18 were goalkeepers (11.2%), 50 were defenders (31.1%), 43 were midfielders (26.6%) and 50 were forwards (31.1%).

All the professional football players were in good hydration condition, both before and after the creatine supplementation protocol (1016.52 ± 4.15 vs. 1017.22 ± 3.88, respectively; *p* = 0.854).

### 3.1. Body Mass Index (BMI)

Of the 161 professional football players, 41 players (25.5%) had a positive adaptation according to the SWC value (0.168 kg/m^2^) in BMI, responding after the creatine supplementation protocol, whereas 120 players were non-responders.

**Figure 1:**
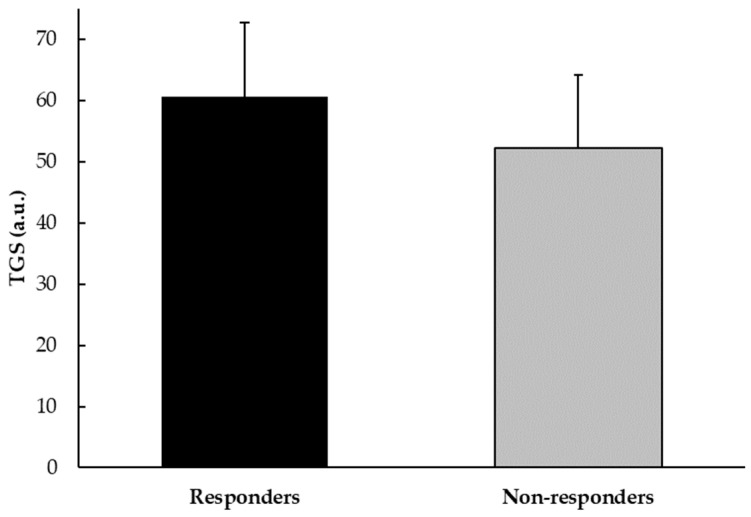
TGS value in BMI responders and non-responders among professional football players.

When aggregating the genotype scores for all polymorphisms, the average total genotype score (TGS) for players who responded with an increase in BMI was 60.57 a.u. (±12.22 a.u.), with a statistical kurtosis of −0.72 (±0.66). For players who did not respond with an increase in BMI, the TGS was 52.28 a.u. (±11.83 a.u.), with a statistical kurtosis of −0.66 (±0.53). The difference in TGS values between responders and non-responders in BMI after creatine supplementation was statistically significant (*p* < 0.001) (Figure 1).

**Figure 2:**
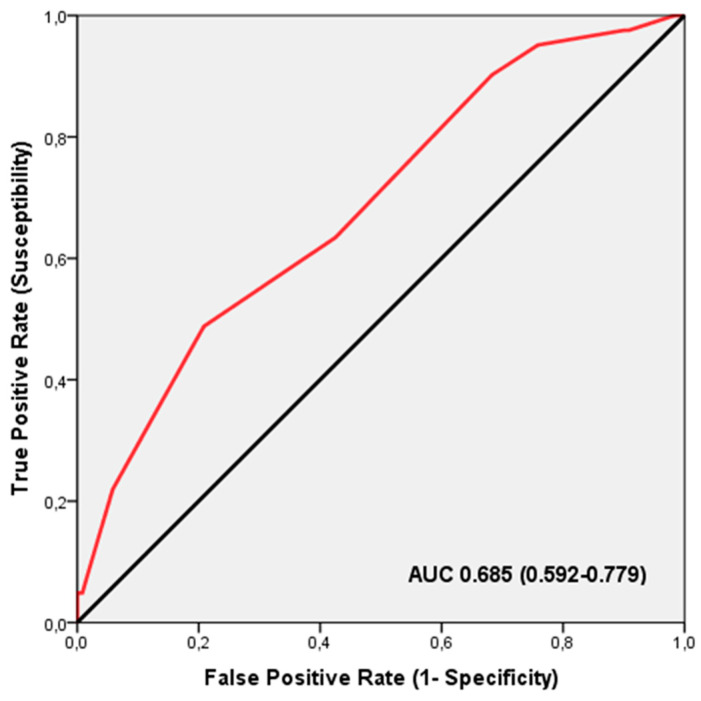
The receiver operating characteristic (ROC) curve illustrates the effectiveness of the TGS in identifying professional football players who are likely to experience an increase in BMI after creatine supplementation, based on their muscle performance profile. AUC, area under the curve.

ROC analysis demonstrated that the TGS had a significant discriminatory power in identifying BMI increases among professional football players undergoing creatine supplementation (AUC = 0.685; 95% CI: 0.592–0.779; *p* = 0.001), with sensitivity at 0.951 and specificity at 0.758 (Figure 2). The TGS threshold for this distinction was 41.66 a.u. Binary logistic regression revealed that players with a TGS above 41.66 a.u. had an odds ratio (OR) of 4.287 (95% CI: 1.426–12.890; *p* = 0.010) for being responders with increased BMI after the creatine supplementation, compared to those with a TGS below this threshold.

The distribution of genotypes related to muscle performance genes revealed statistically significant differences when comparing responders and non-responders in terms of BMI increase. For the *ACTN3* c.1729C>T polymorphism, a significant difference was observed (*p* = 0.046), with a higher frequency of the ‘optimal’ genotype (CC) in BMI responders (46.3%) compared to non-responders (26.7%). Similarly, significant differences were noted for the *CKM* c.*800A>G polymorphism (*p* = 0.044), where the ‘optimal’ genotype (GG) was more prevalent among players with increased BMI (22.0%) versus those without (8.3%). The *MLCK* c.37885C>A polymorphism also showed significant differences, with a higher frequency of the ‘optimal’ genotype (AA) in BMI responders (7.3%) compared to non-responders (0.0%). Although the *AMPD1* c.34C>T polymorphism did not reach statistical significance, it demonstrated a trend (*p* = 0.082) with a greater frequency of the ‘optimal’ genotype (CC) in BMI responders (78.0%) compared to non-responders (59.2%). No significant differences were found for other muscle performance polymorphisms between players who experienced an increase in BMI and those who did not following creatine supplementation (Table 4).

### 3.2. Fat Mass

Thirty-six football players (11.2%) were responders with a decrease in fat mass after the creatine supplementation protocol according to the SWC value (0.218%), whereas 125 players were non-responders, with an increase in fat mass.

In the analysis of genotype scores across all polymorphisms, players who responded with a decrease in fat mass following creatine supplementation had a mean TGS of 57.40 a.u. (±13.02 a.u.), with a statistical kurtosis of −0.40 (±0.62). By contrast, non-responders had a mean TGS of 53.53 a.u. (±12.17 a.u.), with a statistical kurtosis of −0.55 (±0.47). The difference in TGS values between responders and non-responders in fat mass decrease was not statistically significant (*p* = 0.100).

**Figure 3:**
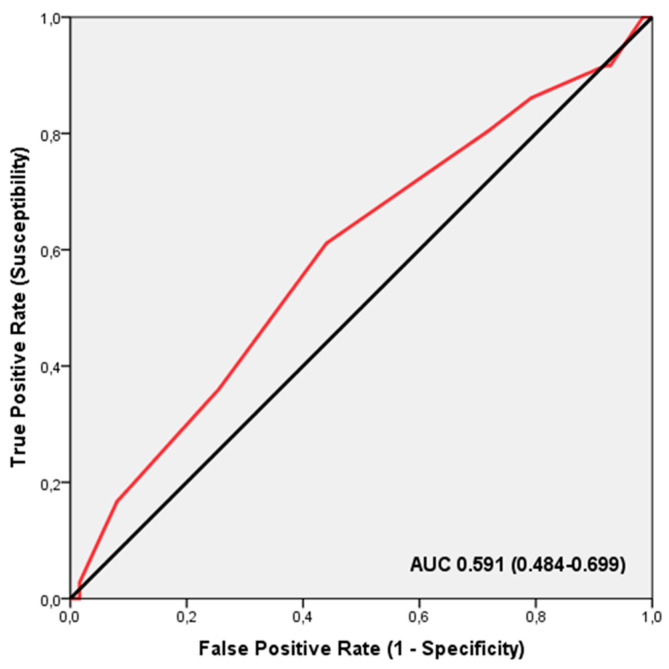
The receiver operating characteristic (ROC) curve illustrates the effectiveness of the TGS in identifying professional football players who are likely to experience a decrease in fat mass following creatine supplementation, based on their muscle performance profile. AUC, area under the curve.

ROC analysis revealed that the TGS did not significantly distinguish between different injury locations in professional football players (AUC = 0.591; 95% CI: 0.484–0.699; *p* = 0.096), with sensitivity at 0.611 and specificity at 0.440 (Figure 3). The TGS threshold for this analysis was 54.16 a.u. Binary logistic regression indicated that players with a TGS above 54.16 a.u. had an odds ratio (OR) of 2.012 (95% CI: 0.938–4.266; *p* = 0.073) for a decrease in fat mass following creatine supplementation, compared to those with a TGS below this threshold.

Genotype distribution of muscle performance genes in the football players with responders of fat mass, when compared to non-responders after the creatine supplementation protocol, did not show differences in any of the polymorphisms (Table 5).

### 3.3. Muscle Mass

Sixty-eight professional football players (42.2%) were responders in muscle mass increase after the creatine supplementation protocol according to the SWC value (0.521%), whereas 93 players (57.8%) were non-responders.

**Figure 4:**
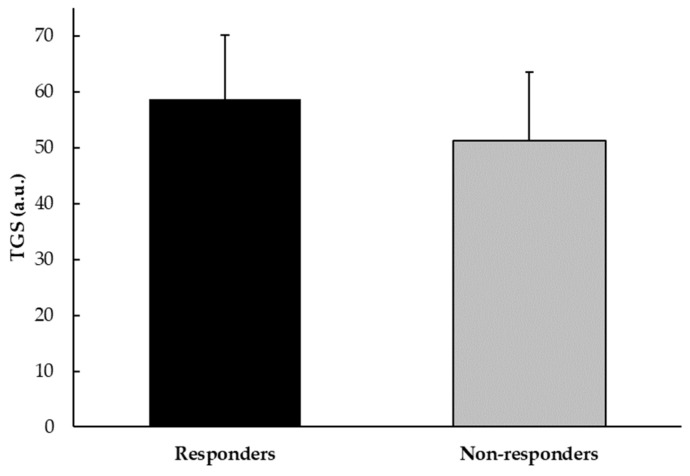
TGS value in muscle mass responders and non-responders among professional football players.

For the genotype scores of all polymorphisms, players who showed an increase in muscle mass had a mean TGS of 58.69 a.u. (±11.38 a.u.), with a statistical kurtosis of −0.23 (±0.55). By contrast, non-responders had a mean TGS of 51.25 a.u. (±12.28 a.u.), with a statistical kurtosis of −0.56 (±0.47). The difference in TGS values between responders and non-responders in muscle mass increase among professional football players was statistically significant (*p* < 0.001) (Figure 4).

**Figure 5:**
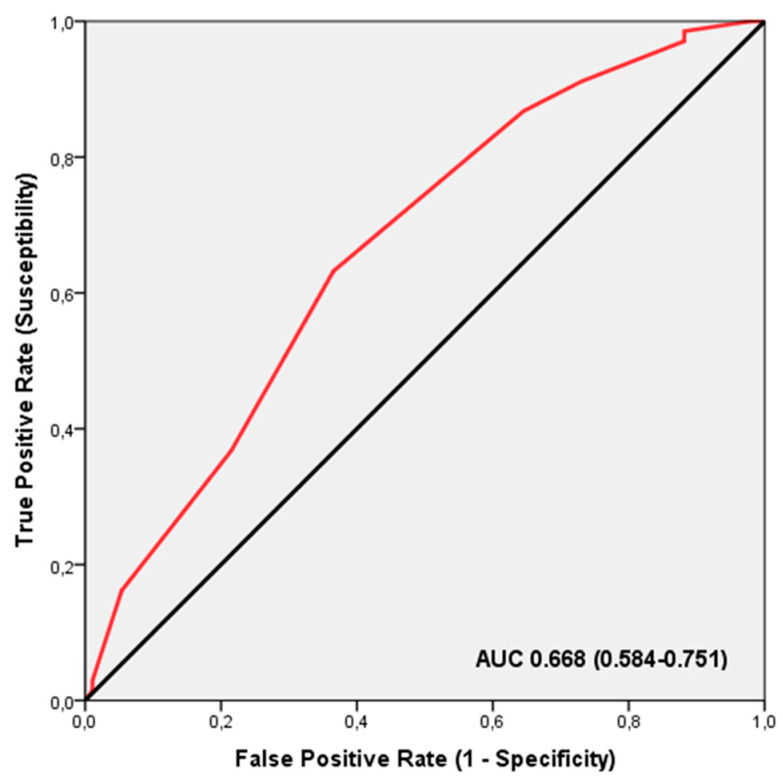
The receiver operating characteristic (ROC) curve summarises how well the TGS identifies professional football players likely to show an increase in muscle mass following creatine supplementation, based on their muscle performance profile. AUC, area under the curve.

ROC analysis demonstrated that the TGS effectively distinguished between professional football players who experienced an increase in muscle mass following creatine supplementation (AUC = 0.668; 95% CI: 0.584–0.751; *p* < 0.001), with a sensitivity of 0.632 and specificity of 0.366 (Figure 5), and other players. The threshold TGS value for this distinction was 54.16 a.u. Binary logistic regression further indicated that players with a TGS exceeding 54.16 a.u. had an odds ratio (OR) of 2.985 (95% CI: 1.560–5.711; *p* = 0.001) for showing a response in muscle mass increase after creatine supplementation, compared to those with a TGS below this threshold.

The distribution of muscle performance gene genotypes revealed statistically significant differences between responders and non-responders in muscle mass increase. For the *AMPD1* c.34C>T polymorphism, responders showed a higher frequency of the ‘optimal’ genotype (CC 77.9%) compared to non-responders (CC 52.7%) (*p* = 0.005). Additionally, the heterozygous genotype (CT) was more common among muscle mass responders (45.2%) than non-responders (20.6%). The *ACE* I/D polymorphism displayed a statistical trend (*p* = 0.065), with a higher frequency of the ‘optimal’ genotype (DD 42.6%) in players who experienced muscle mass increase compared to those who did not (DD 25.8%). No significant differences were observed for other muscle performance polymorphisms between players with increased muscle mass and those without who followed creatine supplementation (Table 6).

### 3.4. Muscle Injuries

Out of the total football players, 92 (57.4%) experienced non-contact muscle injuries following the creatine supplementation protocol during the season.

For BMI, the threshold for a response increase after creatine supplementation was set at a TGS of 41.66 a.u. Binary logistic regression analysis indicated that players with a TGS below 41.66 a.u. had an odds ratio (OR) of 11.435 (95% CI: 3.838–24.067; *p* < 0.001) for sustaining muscle injuries during the season compared to those with a TGS above this threshold.

In terms of decrease in fat mass and muscle mass increase, the response threshold after creatine supplementation was identified as a TGS value of 54.16 a.u. Binary logistic regression analysis revealed that players with a TGS below 54.16 a.u. had an OR of 9.385 (95% CI: 4.535–19.425; *p* < 0.001) for experiencing muscle injuries during the season, relative to those with a TGS above this value.

## 4. Discussion

Although the influence of creatine supplementation and the likelihood of increased body mass have been analysed in several previous investigations, this is the first to investigate the relationship of several polymorphisms of muscle performance-related genes in the association between the responders in BMI, muscle mass increase, and decrease in fat mass after the creatine supplementation protocol and the likelihood of muscle injuries in professional football players. The purpose of this study was to examine the relationship between muscle performance-related genes, creatine supplementation response by BMI, muscle mass increase, and decrease in fat mass and injury risk in men’s professional football players.

The main finding in this study is the correlation between TGS and anthropometric characteristics, showing that ‘optimal’ genotypes of the *ACE* I/D (rs4646994), *ACTN3* c.1729C>T (rs1815739), *AMPD1* c.34C>T (rs17602729), *CKM* c*800A>G (rs8111989), and *MLCK* c.37885C>A (rs2849757) polymorphisms suggest some type of creatine supplementation-related benefit, especially in muscle mass gain and the prevention of muscle injury.

However, players with different genotypes in these polymorphisms showed different characteristics in response to creatine supplementation. (a) Players with the CC genotype of the *AMPD1* and *ACTN3* polymorphisms, GG of the *CKM* polymorphism, and AA genotype of the *MLCK* c.37885C>A polymorphism had a higher response of increased BMI after creatine supplementation, with a higher frequency of response in BMI increase. (b) Players with the DD genotype of the *ACE* polymorphism and CC genotype of the *AMPD1* polymorphism had a higher response of increased muscle mass after creatine supplementation. The CT genotype of the *AMPD1* showed a lower frequency of increased muscle mass response after creatine supplementation. (c) Players with the CT genotype of the *AMPD1* polymorphism had a lower frequency of increased muscle mass after creatine supplementation; and (d) no association was found in the response of fat mass decrease after creatine supplementation in any of the polymorphisms included. Collectively, this information suggests that *ACE*, *ACTN3*, *AMPD1*, *CKM*, and *MLCK* c.37885C>A genotypes affect the likelihood of non-contact injuries during the football season. Furthermore, having a TGS above the cut-off point for being a responder to creatine supplementation is somewhat protective against muscle injury, as is the case for responders in increased BMI, muscle mass increase, and fat mass decrease, while those players who did not respond to creatine supplementation had a higher risk of muscle injuries.

### 4.1. Creatine Supplementation

The use of ergogenic supplements continues to rise annually across both amateur and professional sports, with a particularly high incidence in football. The effectiveness of these supplements is influenced by a combination of the player’s intrinsic factors and environmental conditions [56,57]. Commonly used supplements include caffeine, creatine, protein, carbohydrate and electrolyte drinks, tart cherry juice, beetroot juice rich in nitrates, sodium bicarbonate with minerals, yohimbine, and proprietary nutraceuticals. These supplements are believed to enhance football performance but may also introduce variables that could affect study outcomes [58,59,60,61]. Consequently, evidence regarding the efficacy of dietary supplements for improving football performance remains mixed, limited, or sometimes lacking altogether [20,62]. However, this investigation demonstrates the effectiveness of creatine supplementation in increasing muscle mass in a sample of players with diverse genetic backgrounds and different dietary habits. The clubs involved adhered to the same creatine supplementation protocol as previously presented [22,46], making this a pragmatic study. Despite the similar characteristics of the professional football players, only 11.2% were responders to a decrease in fat mass, 25.5% were responders to an increase in BMI, and 42.2% were responders to a muscle mass increase. This current investigation suggests that genetics may constitute an important contributing factor for a player’s predisposition to respond in BMI and muscle mass increase after creatine supplementation. Additionally, through the assessment of a TGS that includes only six polymorphisms involved in muscle performance, players with a higher predisposition to response after creatine supplementation can be identified and placed in a special programme to enhance their intrinsic nature-associated susceptibility to gain muscle mass that could be associated with performance in professional football and protection against muscle injuries during the season. The knowledge provided by this research with genetic data could constitute a new tool to optimise and individualise the use of supplementation in professional football players alongside established nutritional methodologies. This result not only has important implications for clinical practice but also opens new avenues for future research.

### 4.2. Genetic Profile and Creatine Supplementation

Although excellent muscle performance in elite athletes is facilitated by an optimal polygenic profile [23,24,63,64], the methodological rigour and evidence in genetic association research in football still has room for improvement [65]. This investigation indicates that the combined influence of these polymorphisms is strong enough to link the response after creatine supplementation to muscle performance. Previous research suggests that creatine supplementation can improve strength, power, and work capacity during short, high-intensity periods, which may translate into improved performance on the field [66,67,68]. However, it is important to note that the effects of creatine supplementation may vary depending on an individual’s genetics. Some athletes may experience a more pronounced response to supplementation than others due to differences in muscle creatine storage capacity and the efficiency of energy systems. In turn, some evidence has been shown on creatine supplementation and its effect on sports injuries and recovery [2,69,70,71]. The data reported in this investigation could relate creatine supplementation with the genetic profile in muscle performance and the related benefit in reducing muscle injuries in this cohort of athletes [23]. The identification of genetic markers associated with the regulation of energy metabolism in skeletal muscles can help sports nutritionists and coaches to develop personalised strategies to adapt the nutritional strategies of supplementation protocols according to the professional football player’s genetic profile, previously presented [23,64].

### 4.3. ACE I/D Polymorphism

The *ACE* gene and, specifically, the I/D polymorphism presents evidence of an association with sports performance in strength and power modalities, especially the D allele [25]. The I allele has been consistently demonstrated to be associated with endurance-orientated events. Meanwhile, the D allele is associated with strength- and power-oriented performance [72,73]. The *ACE* I/D polymorphism could significantly influence creatine response and overall athletic performance. Knowing one’s genotype should provide advantages for optimising training and supplementation strategies, helping athletes reach their full potential more effectively. This research confirms that players with the DD genotype had a higher frequency of response to increased muscle mass, data that confirm the D allele as a predictor of performance in power sports modalities associated, in turn, with the response of increased muscle mass under creatine supplementation, presenting as a growth factor [25,74]. The data presented in this investigation should be corroborated by future studies to confirm this gene as a predictor of power due to muscle mass increase.

### 4.4. ACTN3 c.1729C>T Polymorphism

The c.1729C>T polymorphism of the *ACTN3* gene has been one of the most studied in sports performance [40,75] and non-contact muscle injuries [76,77]. In this regard, it has been shown that the C allele is predominant in fast-twitch skeletal muscle fibre and is associated with greater muscle strength [26,72]. This investigation has shown that professional football players with a CC genotype had a higher frequency of response to increase BMI after creatine supplementation. However, there was no association in the response to muscle mass gain after creatine supplementation. Many studies have shown the combination of *ACE* and *ACTN3* polymorphisms in muscle strength gain and power in football [43,78,79], and this could be one of the associations of this increase in BMI and muscle mass in response to sports performance. Finally, this investigation demonstrates the influence of the *ACTN3* gene on the response to increase BMI in professional football players.

### 4.5. AMPD1 c.34C>T Polymorphism

A new genetic target involved in muscle metabolism, the c.34C>T polymorphism of the *AMPD1* gene, associated with cramps, early fatigue [80], and non-contact muscle injuries in football [23], has been discovered in recent years. Muscle adenosine triphosphate (ATP) is critical to athletic performance. The results presented in this research show players with the CC genotype with a higher frequency of responding to an increase in muscle mass due to muscle mass gain, data that confirm the individual data for *ACE* and *ACTN3* polymorphisms. With the data presented in this study, the author concludes that the cause of response to increase muscle mass and BMI under creatine supplementation is due to the capability of muscle cells to optimise energy in football because of the c.34C>T polymorphism of the *AMPD1*. This investigation shows that the *AMPD1* gene is a relevant factor for professional football players’ performance and status [24,64] and protects them from injuries, especially those of a musculoskeletal nature [23].

### 4.6. CKM c*800A>G Polymorphism

The c*800A>G polymorphism in the *CKM* gene plays an important role in energy provision during high-intensity muscle contraction and sports performance [32]. It has been shown that the G allele and GG are higher in power modalities [32]; however, there is still controversy over the association of this polymorphism in power athlete status [81,82]. The main outcomes of this research indicate that football players with the GG had a higher response to increased muscle mass and BMI under creatine supplementation, showing new data about the relevance of the GG in power athlete status. However, these results should be interpreted with caution, suggesting the need for further studies in which more factors indicating how the *CKM* gene is related to performance can be analysed—more so in football performance, where the association of the c.*800A>G polymorphism in muscle, tendon, and ligament injuries has recently been shown [31,83].

### 4.7. MLCK c.49C>T and c.37885C>A Polymorphisms

In the MLCK gene, two specific polymorphisms have been linked to strength loss following exercise [33,84] and are crucial for regulating smooth muscle contraction [85,86]. Research indicates that individuals with the CA genotype for the c.37885C>A polymorphism may experience greater exercise-induced muscle damage [33]. Conversely, the AA genotype was found to be advantageous for increasing BMI after creatine supplementation compared to the CA and CC genotypes among professional football players (Table 5). This suggests that optimal muscle contraction and reduced strength loss during exercise are associated with the AA genotype, which may contribute to improved BMI response. The A allele is linked to maintaining strength during athletic activities, as noted in previous studies [23], though these findings will be further validated in subsequent research with this athlete cohort. For the c.49C>T polymorphism, no associations were found with the analysed anthropometric profiles, aligning with earlier studies that reported no link between this polymorphism and exertional muscle damage or injuries in professional football players [23].

### 4.8. Association of TGS with BMI, Muscle Mass Response, and Non-Contact Musculoskeletal Injuries in Professional Football Players

Indeed, the TGS of these polymorphisms involved in muscular performance shows that the likelihood of sustaining a non-contact musculoskeletal injury in professional football players was associated with BMI and muscle mass responders. Non-contact musculoskeletal injuries presented a higher risk in the TGS <41.66 a.u. football players than in their >41.66 a.u. counterparts for BMI responders and a higher risk in the <54.16 a.u. football players than in their >54.16 a.u. counterparts for muscle mass responders. This information from genetic profile studies using TGS should be implemented in the field of genetics in the future, as they provide more complete information to define a phenotype in the field of nutrition and supplementation optimisation, injuries [23,83,87], and even performance [24,87,88] than previous monogenic studies [40,76,89]. The results of this study will allow for specific programmes for the individualisation of nutrition and supplementation strategies, and the use of this genetic profile deserves further investigation.

To integrate the metabolic demands of a player’s position with the metabolic role of creatine and the potential benefits of its supplementation, different positions in football have varying metabolic demands. Midfielders generally cover the most distance during a game, requiring a blend of aerobic endurance and anaerobic capacity. Forwards and wingers need explosive power and speed for short bursts to make plays and score goals. Defenders require strength and agility to tackle and intercept, along with anaerobic endurance for quick recoveries [20,90]. Creatine is a naturally occurring compound that helps regenerate ATP, the primary energy currency in cells, especially during high-intensity, short-duration activities [71,91]. This is particularly relevant in football, where players often perform sprints, jumps, and rapid direction changes.

The biological mechanisms underlying the effects of creatine supplementation are influenced by specific genetic polymorphisms. For instance, polymorphisms in the *CKM* gene can affect creatine kinase enzyme activity, which is crucial for the regeneration of ATP. Variations in this gene might result in differing responses to creatine supplementation, impacting muscle strength and recovery [22]. Similarly, polymorphisms in the solute carrier family 22 member 4 (*SLC22A4*) gene can affect the transport and uptake of creatine into muscle cells, potentially influencing the efficacy of supplementation [92]. Additionally, genetic variations in *ACTN3* have been associated with differences in muscle performance and recovery [89], which could modulate the benefits derived from creatine. Understanding these genetic factors can help tailor creatine supplementation to individual players based on their genetic profiles, optimising the benefits for their specific metabolic needs. Incorporating creatine supplementation into the diet of football players can, therefore, support the specific metabolic demands of their positions, enhancing overall performance and endurance in relation to their genetic profile information.

### 4.9. Limitations

Despite its strengths, this study has several limitations; these include the following: (i) factors such as playing position and other intrinsic characteristics of professional football players were not assessed, which could have impacted this study’s outcomes; (ii) although the sample size of the professional football players was limited, the cohort studied is representative; (iii) the absence of a placebo control and randomization prevents the confirmation of changes in BMI, muscle mass, and fat mass independently of the creatine supplementation protocol; (iv) adherence to the supplementation protocol should be more rigorously monitored, potentially through daily checks or electronic reminders, to reduce participant exclusion; (v) future research may uncover genetic variants not included in this study’s model, which could explain individual differences in response to sports supplementation and performance; (vi) more longitudinal studies involving larger cohorts of professional football players, including female players, are needed; as it is premature to rely on genetic testing to predict responses to creatine supplementation and associated injury risks in professional football, (vii) future studies should consider the standard ergometric data of professional football players, including VO_2_max, sprint performance, and countermovement jump (CMJ), to establish associations with their genetic profiles; and (viii) this study does not show the performance data of professional football players, suggesting future studies where changes in body composition could be associated with performance.

This is the first study to demonstrate that a genetic profile influences the response of professional football players to muscle mass gain and injury reduction under creatine supplementation. Further studies could help develop genetic profiling models as a potential tool to help predict the effect of supplementation in these football players. Indeed, the genes positively associated with muscle performance and responses related to increases in BMI and muscle mass included *ACE*, *ACTN3*, *AMPD1*, *CKM*, and the *MLCK* c.37885C>A polymorphisms. Among these, the c.34C>T polymorphism in the *AMPD1* gene was uniquely linked to muscle response to creatine supplementation in professional football players. This highlights AMPD1 as a crucial gene for understanding optimal muscle performance in football, given its role in enhancing muscle energy, as noted in previous studies [23,63,83]. Additionally, this study underscores the importance of incorporating epigenetic and environmental factors when analysing elements associated with football performance related to muscle metabolism. Such an approach could deepen our understanding of the interactions between genetics, ergogenic aid supplementation, and injury risks [64].

This study brings a new dimension to scientific knowledge by combining genetics with sports nutrition and injury prevention. By analysing, for the first time, the relationship between muscle performance-related genes and creatine supplementation in professional football players and by demonstrating the feasibility of a total genotype score, this study opens new avenues for future research and practical applications in elite sports.

## 5. Conclusions

This study is the first to demonstrate that the response to increased muscle mass under creatine supplementation in professional football players is associated with a genetic score obtained from the combination of favourable/unfavourable genotypes in genes involved in muscle performance. The CC genotype and C allele of *AMPD1* appear to be the polymorphism that best correlate with a higher probability of response under creatine supplementation to increase BMI and muscle mass in the cohort of professional football players studied.

## Figures and Tables

**Table 1:** Professional football players’ baseline characteristics.

	Professional Football Players, *n* = 161
Age, mean (SD)	26.44 (4.54)
Weight, mean (SD)	71.64 (7.23)
Height, mean (SD)	180.71 (6.12)
BMI, mean (SD)	21.92 (1.75)
Fat mass %, mean (SD)	7.02 (1.12)
Muscle mass %, mean (SD)	47.65 (1.24)

SD: standard deviation.

**Table 2:** Genomic location and minor allele frequency (MAF) for selected genes in muscle performance.

Symbol	Gene	dbSNP	Genomic Location	MAF Football Players	MAF (IBS) *	HWE	FIS
*ACE*	Angiotensin-converting enzyme	rs4646994	17q23.3	42.5% (I)	36.7% (I) **	*p* = 0.253	−0.32
*ACTN3*	Alpha-actinin-3	rs1815739	11q13.2	45.3% (T)	43.9% (T)	*p* = 0.789	−0.04
*AMPD1*	Adenosine monophosphate deaminase 1	rs17602729	1p13.2	18.6% (T)	14.0% (T)	*p* = 0.587	−0.18
*CKM*	Muscle-specific creatine kinase	rs8111989	19q13.32	35.7% (G)	26.6% (G)	*p* = 0.101	−0.34
*MLCK*	Myosin light-chain kinase	rs2700352	3q21.1	28.5% (T)	20.1% (T)	*p* = 0.089	−0.46
	Myosin light-chain kinase	rs28497577	3q21.1	21.7% (A)	10.3% (A)	*p* = 0.063	−0.57
*Overall SNPs*						*p* = 0.268	−0.31

IBS, Iberian population in Spain * [44] ** [45]; FIS, inbreeding coefficient; HWE, Hardy–Weinberg equilibrium; MAF, minor allele frequency; SNP, single-nucleotide polymorphism.

**Table 3:** Genotype distribution in the professional football players.

Symbol	Gene Name	Polymorphism	dbSNP	Genotype Score	Professional Football Players
*ACE*	Angiotensin-converting enzyme	I/D	rs4646994	2 = DD	53 (32.9%)
				1 = ID	79 (49.1%)
				0 = II	29 (18.0%)
*ACTN3*	Alpha-actinin-3	c.1729C>T	rs1815739	2 = CC	51 (34.6%)
				1 = CT	74 (46.0%)
				0 = TT	36 (22.4%)
*AMPD1*	Adenosine monophosphate deaminase 1	c.34C>T	rs17602729	2 = CC	103 (64.0%)
				1 = CT	56 (34.8%)
				0 = TT	2 (1.2%)
*CKM*	Muscle-specific creatine kinase	c.*800A>G	rs8111989	2 = GG	19 (11.8%)
				1 = GA	77 (47.8%)
				0 = AA	65 (40.4%)
*MLCK*	Myosin light-chain kinase	c.49C>T	rs2700352	2 = CC	77 (47.8%)
				1 = CT	76 (47.2%)
				0 = TT	8 (5.0%)
	Myosin light-chain kinase	c.37885C>A	rs28497577	2 = AA	3 (1.9%)
				1 = CA	64 (39.8%)
				0 = CC	94 (58.4%)

**Table 4:** Genotype distribution among professional football players in BMI responders and non-responders.

Symbol	Gene	Polymorphism	dbSNP	Genotype Score	Responders BMI	Non-Responders BMI	*p* Value
*ACE*	Angiotensin-converting enzyme	I/D	rs4646994	2 = DD	18 (43.9%)	35 (29.2%)	0.205
				1 = ID	16 (39.0%)	63 (52.5%)	
				0 = II	7 (17.1%)	22 (18.3%)	
*ACTN3*	Alpha-actinin-3	c.1729C>T	rs1815739	2 = CC	19 (46.3%) ^↑^	32 (26.7%) ^↓^	0.046
				1 = CT	13 (31.7%)	61 (50.8%)	
				0 = TT	9 (22.0%)	27 (22.5%)	
*AMPD1*	Adenosine monophosphate deaminase 1	c.34C>T	rs17602729	2 = CC	32 (78.0%) ^↑^	71 (59.2%) ^↓^	0.082
				1 = CT	9 (22.0%)	47 (39.2%)	
				0 = TT	0 (0.0%)	2 (1.7%)	
*CKM*	Muscle-specific creatine kinase	c.*800A>G	rs8111989	2 = GG	9 (22.0%) ^↑^	10 (8.3%) ^↓^	0.044
				1 = GA	19 (46.3%)	58 (48.4%)	
				0 = AA	13 (31.7%)	52 (43.3%)	
*MLCK*	Myosin light-chain kinase	c.49C>T	rs2700352	2 = CC	23 (56.1%)	54 (45.0%)	0.457
				1 = CT	16 (39.0%)	60 (50.0%)	
				0 = TT	2 (4.9%)	6 (5.0%)	
	Myosin light-chain kinase	c.37885C>A	rs28497577	2 = AA	3 (7.3%) ^↑^	0 (0.0%) ^↓^	0.010
				1 = CA	17 (41.5%)	47 (39.2%)	
				0 = CC	21 (51.2%)	73 (69.8%)	

↑: statistical higher frequency; ↓: statistical lower frequency.

**Table 5:** Genotype distribution among professional football players in fat mass responders and non-responders.

Symbol	Gene	Polymorphism	dbSNP	Genotype Score	Responders’ Fat Mass	Non-Responders’ Fat Mass	*p* Value
*ACE*	Angiotensin-converting enzyme	I/D	rs4646994	2 = DD	17 (47.2%)	36 (28.8%)	0.099
				1 = ID	15 (41.7%)	64 (51.2%)	
				0 = II	4 (11.1%)	25 (20.0%)	
*ACTN3*	Alpha-actinin-3	c.1729C>T	rs1815739	2 = CC	14 (38.9%)	37 (29.6%)	0.531
				1 = CT	14 (38.9%)	60 (48.0%)	
				0 = TT	8 (22.2%)	28 (22.4%)	
*AMPD1*	Adenosine monophosphate deaminase 1	c.34C>T	rs17602729	2 = CC	25 (69.4%)	78 (62.4%)	0.417
				1 = CT	10 (27.8%)	46 (36.8%)	
				0 = TT	1 (2.8%)	1 (0.8%)	
*CKM*	Muscle-specific creatine kinase	c.*800A>G	rs8111989	2 = GG	4 (11.1%)	15 (12.0%)	0.793
				1 = GA	19 (52.8%)	58 (46.8%)	
				0 = AA	13 (36.1%)	52 (41.6%)	
*MLCK*	Myosin light-chain kinase	c.49C>T	rs2700352	2 = CC	19 (52.8%)	58 (46.4)%	0.752
				1 = CT	15 (41.7%)	61 (48.8%)	
				0 = TT	2 (5.6%)	6 (4.8%)	
	Myosin light-chain kinase	c.37885C>A	rs28497577	2 = AA	2 (5.6%)	1 (0.8%)	0.140
				1 = CA	12 (33.3%)	52 (41.6%)	
				0 = CC	22 (61.1%)	72 (57.6%)	

**Table 6:** Genotype distribution among professional football players in muscle mass responders and non-responders.

Symbol	Gene	Polymorphism	dbSNP	Genotype Score	Responders Muscle Mass	Non-Responders Muscle Mass	*p* Value
*ACE*	Angiotensin-converting enzyme	I/D	rs4646994	2 = DD	29 (42.6%) ^↑^	24 (25.8%) ^↓^	0.065
				1 = ID	30 (44.1%)	49 (52.7%)	
				0 = II	9 (13.2%)	20 (21.5%)	
*ACTN3*	Alpha-actinin-3	c.1729C>T	rs1815739	2 = CC	27 (39.7%)	24 (25.8%)	0.106
				1 = CT	30 (44.1%)	44 (47.3%)	
				0 = TT	11 (16.2%)	25 (26.9%)	
*AMPD1*	Adenosine monophosphate deaminase 1	c.34C>T	rs17602729	2 = CC	53 (77.9%) ^↑^	50 (52.7.8%) ^↓^	0.005
				1 = CT	14 (20.6%) ^↓^	42 (45.2%) ^↑^	
				0 = TT	1 (1.5%)	1 (1.1%)	
*CKM*	Muscle-specific creatine kinase	c.*800A>G	rs8111989	2 = GG	11 (16.2%)	8 (8.6%)	0.194
				1 = GA	34 (50.0%)	43 (46.2%)	
				0 = AA	23 (33.8%)	42 (45.2%)	
*MLCK*	Myosin light-chain kinase	c.49C>T	rs2700352	2 = CC	34 (50.0%)	43 (46.2%)	0.880
				1 = CT	31 (45.6%)	45 (48.4%)	
				0 = TT	3 (4.4%)	5 (5.4%)	
	Myosin light-chain kinase	c.37885C>A	rs28497577	2 = AA	1 (1.5%)	2 (2.2%)	0.790
				1 = CA	29 (42.6%)	35 (37.6%)	
				0 = CC	38 (55.9%)	56 (60.2%)	

↑: statistical higher frequency; ↓: statistical lower frequency.

## Data Availability

The original contributions presented in this study are included in the article further inquiries can be directed to the corresponding author.
